# Academic entrepreneurial engagement for frugal innovation in higher education institutions: a systematic literature review

**DOI:** 10.12688/f1000research.73312.1

**Published:** 2021-10-25

**Authors:** Oluwayomi Toyin Ojo, Magiswary Dorasamy, Melissa W. Migin, Jayamalathi Jayabalan, Rajeswari R, Soon Seng Tung

**Affiliations:** 1 Faculty of Management, Multimedia University, Cyberjaya, Selangor, 63100, Malaysia; 2 Faculty of Accountancy and Management, Universiti Tunku Abdul Rahman, Kajang, Selangor, 43000, Malaysia; 3 Jai Bharath School of Management Studies, Perumbavoor, Kerala, 683562, India; 4 Alibaba Group Malaysia Sdn Bhd, Kuala Lumpur, Wilayah Persekutuan, 52900, Malaysia

**Keywords:** Academic entrepreneurial engagement, higher education institutions, Frugal innovation, Malaysia, Private university, Sustainability, Game changer, Systematic literature review

## Abstract

Higher education institutions (HEI) are faced with increasing challenges related to shrinking resources, high operation costs, the COVID-19 pandemic, decreasing student enrolment rates, and pressure to contribute to regional development and economic growth. To overcome such challenges, academics must move beyond their traditional functions of research and teaching and engage in entrepreneurial activities. Through engagement in entrepreneurial activities, academics can contribute to frugal innovation (FI) in private HEI (PHEI). The literature in this context emphasizes that academic entrepreneurial engagement (AEE) will lead to innovation, the identification of opportunities for new business ventures, financial rewards for institutions and academics, an impact on the economy, and the enhancement of social welfare. This study presents a systematic review of the literature and adopts the Transfield five-phase strategy to review the literature on AEE from the past two decades (2000–2020). A total of 1,067 papers on FI are obtained, only five of which focus on AEE. Moreover, papers related to AEE for FI are few. The study presents the research gaps, challenges, and potential factors for further research in this context. We conclude that FI for AEE in PHEI can be a game-changer for future sustainability. Moreover, we believe that the outcome of this review warrants further research.

## Introduction

Private higher education institutions (PHEI) play an important role in a nation’s economic development and prosperity by supplying skilled workforce and generating basic knowledge for solving societal problems. Notwithstanding their strategic contributions, PHEI are challenged by dwindling resources and the need to contribute to the economy and social development. ^1^ For instance, in Malaysia, PHEI are faced with rising operating costs, pressure to increase their global ranking, problems in balancing teaching and research task allocation, decreasing government funding and shrinking budg *et al*locations for staff development and empowerment programmes. ^2^

Many PHEI in Malaysia incurred losses for years and are unable to access adequate capital. ^3^ A recent report revealed that 55% of Malaysia’s PHEI incurred losses, and approximately 44% were financially insolvent. As a result, more than 5,800 academics are faced with an uncertain career outlook, and approximately 121,000 students are at risk of receiving poor-quality education. ^4^ Besides, PHEI depend mainly on student fees to offset their operating costs. However, funding sources continue to decline. The annual budget of the National Higher Education Fund Corporation, which is a major provider of educational loans in Malaysia, was severely reduced owing to the high default and low repayment rates of existing loans. ^4^ Thus, to survive, PHEI management must devise effective strategies to attain financial stability amidst dwindling resources.

Furthermore, the COVID-19 pandemic created additional challenges and heightened the impact of existing problems encountered by PHEI. The Movement Control Order (MCO) and border closure policy to curtail the spread of COVID-19 resulted in a drop in student enrolment. Specifically, international student enrolment declined drastically owing to students’ inability to gain entry into the country. ^5^ Prospective students also deferred or delayed their studies, whereas others were unable to pay tuition fees owing to loss of jobs and the economic recession. Inevitably, this situation will further put a strain on the cash flow of institutions ^5^ and the economy. These challenges indicate the urgent need for PHEI management to restrategise to enhance their output and quality amidst dwindling resources.

In coping with the challenges associated with resource constraints and adversities, academics are expected to venture beyond their traditional responsibilities of conducting research, accomplishing administrative tasks and performing teaching activities and engage in entrepreneurial activities. ^6^ By fostering entrepreneurial practices among academics, PHEI can stimulate innovative outcomes to improve economic growth, create jobs and research opportunities for graduates and support the educational ecosystem. ^7^ PHEI establish initiatives to promote educational ecosystems that are conducive to entrepreneurial activity for academics. ^8^^–^^10^ Hence, researchers paid attention to factors influencing university faculty members’ entrepreneurial intention, motivation and behaviour in intrapreneurial activities. ^11^ Nevertheless, insights into the antecedents of academic entrepreneurial engagement (AEE) to enhance innovative performance are limited.

Early attempts to promote academic engagement in entrepreneurial activities can be traced back to the policy initiatives of the Bayh–Dole Act in the United States in 1980, which aimed to contribute to economic development and promote commercialisation and other forms of university technology transfer. ^12^ Recent studies on AEE focused on different types of engagement, i.e. consultations, collaborations, contract research, training joint publications, advisory board membership, industry findings, conferences and so on. ^13^ Moreover, researchers examined the determinants and outcomes of academic entrepreneurial intention. ^14^ Academic entrepreneurial intention is underscored by capability factors such as entrepreneurial-related abilities, education, human capital and industrial experience. ^14^

### Academic Entrepreneurial Engagement (AEE)

AEE entails the commercialisation of scientific knowledge, including the transformation of knowledge into products and processes that may inevitably contribute to economic growth and innovation. AEE refers to academic engagement in activities beyond the traditional functions of research, administration and teaching, including formal and informal activities, such as collaborations with businesses and industries, new-firm creation, research output patenting, invention disclosures by academics to technology transfer offices, academic knowledge transfer, research output licensing, contract research, consulting, research collaborations, knowledge transfer mechanisms, student placements, training and continued professional development, leading to financial rewards for individual academics and institutions. ^15^ Academic engagement in entrepreneurial activities supports knowledge transfer and technology commercialisation and can contribute significantly to entrepreneurial ecosystem development.

PHEI are faced with increasing challenges related to shrinking budgets, adapting to changing contexts and contributing to economic development and competitiveness. Such challenges encourage universities to embrace significant transformation towards becoming an entrepreneurial entity. ^14^ To overcome challenges, considerable pressure is placed on academics to become entrepreneurial. The literature ascertains the role of individual academics ^16^ through their effective transfer of knowledge to form new ideas and inventions that can help universities achieve their entrepreneurial mission and promote sustainable development.

Current studies on AEE focused on factors shaping academic entrepreneurial intention, exploring individual, organisational and institutional variables. ^17^^,^^18^ However, social recognition, peer pressure and academic effective duty were identified as factors influencing academic entrepreneurial intention. ^13^ Previous research explored various academic forms of engagement, such as spinoffs, firm creation, licences and joint ventures and so on. ^17^^,^^18^ Other studies investigated factors motivating individual academics to start a business, such as their work routine, need for independence and desire to become wealthy.

Previous studies indicated that AEE is essential for creating an entrepreneurial university. ^19^^–^^21^ Academic engagement in entrepreneurial activities can help enhance university global performance through frugal innovation (FI). FI may be a game changer for PHEI to leapfrog into the arena of effective academic entrepreneurial strategies based on limited resources. Hence, examining the potential of AEE to support PHEI is crucial.

### Frugal Innovation (FI)

FI is also known as *jugaad* innovation, which demands rapid and rational adaptability to changing conditions. ^22^^,^^23^ FI changed the nature of innovation as the ‘ability to accomplish more with less’, resulting in increased economic and societal value whilst reducing resource consumption. ^23^ The main characteristics of FI are based on cost dimensions, such as reducing costs or purchase prices and the overhead on nonessential activities, increasing ‘value proposition’, decreasing unnecessary functions and features that are non-value adding and minimising the use of resources. ^24^^,^^25^ Therefore, FI enables the significant reduction in resource consumption whilst achieving superior-quality standards and considering the goal of creating a frugal environment. The concept of FI requires further in-depth research using conceptual and empirical techniques.

In higher learning institutions (HLIs), some academicians and management personnel emphasise teaching and learning, student services and community participation, whereas others are concerned with achieving national goals by developing industry-oriented technical innovation, interacting with businesses and engaging in technology transfer. Clearly, HLIs contribute by engaging in R&D collaboration, skills development training, commercialisation through spinoffs and the co-creation of knowledge and ideas and entrepreneurial and technical expertise to overcome community challenges and attain national goals. Hence, emphasis is placed on exploring FI principles as a game changer for the sustainability of PHEI.

This study will provide academic researchers with insights into what has been done with regard to FI for AEE and PHEI to prioritise the factors that can influence AEE for FI in PHEI.

Given this background, the research questions of this study are as follows:

1. Does a research gap exist in AEE studies to support FI pertaining to motivation, opportunity and the ability of academics in PHEI?
2. What are the challenges encountered by academics and the key factors influencing the pursuit of entrepreneurial initiatives in PHEI?
3. Can AEE for FI be a game changer for supporting constraints in PHEI?

The objectives of this proposal are as follows:

1. To identify the research gaps in studies on FI for AEE pertaining to motivation, opportunity and the ability of academics in PHEI
2. To investigate the challenges faced by academics in pursuing entrepreneurial initiatives in PHEI
3. To explore the role of FI in AEE as a game changer for PHEI

We examine 1,067 papers on FI for AEE in PHEI and identify four major gaps in the literature. We believe that our findings warrant the attention of the research community.

## Methods

**Figure 1.**
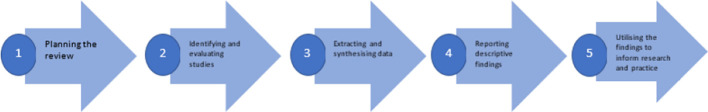
Systematic literature review stages.

This paper was designed to present the identified research gaps and insights to examine FI as a game changer for AEE. Based on the literature, key factors influencing AEE to achieve FI in HLIs were explored. This literature review was based on the five stages of the systematic review as illustrated in Figure 1 proposed by Tranfield et al., 2003. ^26^

### Stage 1. Planning the review

This review aims to identify the research gaps, challenges and factors supporting FI for AEE in PHEI. In addition, this review aims to offer researchers a comprehensive review of previous works related to FI to support AEE initiatives, specifically in the form of a conceptual framework. Finally, the outcome of this review process will offer academic entrepreneurial communities a series of research ideas to advance the field.

### Stage 2. Identifying and evaluating studies

In this stage, papers were examined for the selected keywords. The keywords included general key terms (‘AEE’) regarding specific keywords (‘FI for AEE in PHEI’). The focus of this review is to analyse research on AEE in PHEI. The factors and challenges mentioned in the literature were further classified into several categories.

*Inclusion and exclusion criteria*

**Figure 2.**
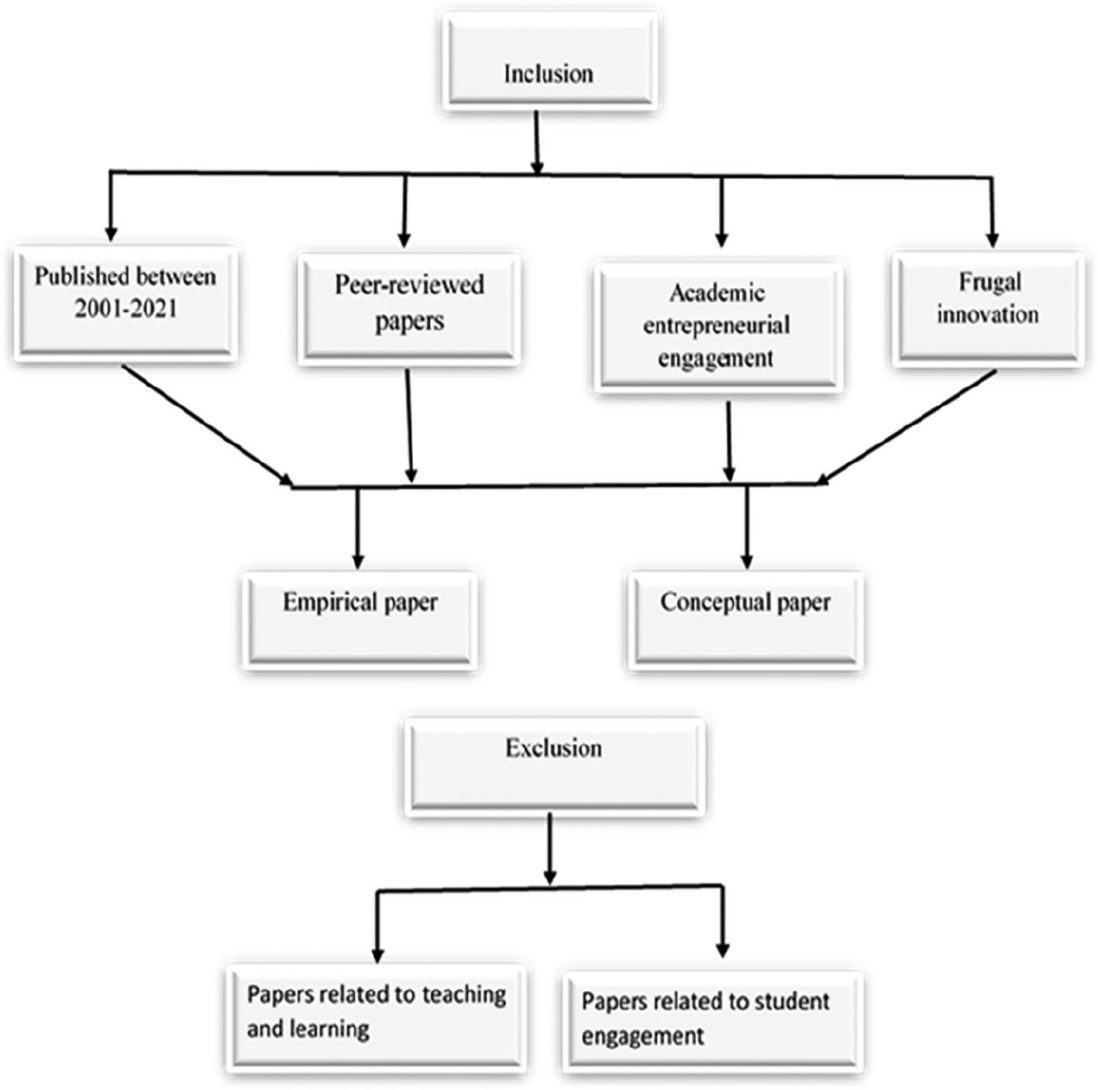
Inclusion and exclusion criteria.

Figure 2 shows the inclusion and exclusion criteria for selecting the papers. The selected papers were those published in the past two decades, peer reviewed, related to AEE focusing on FI and of scholarly origin and either conceptual or empirical papers.

*Keywords*

We focused on six major research areas, namely, (1) AEE, (2) FI, (3) institutions of higher learning (IHL), (4) motivation, (5) ability and (6) opportunity. For the first area, we included terms such as ‘Academic Entrepreneurial Engagement’ and ‘Frugal Innovation’. Each keyword set was combined with other keywords. Table 1 presents the keyword sets used for this research

**Table 1.** Keyword combinations.

Keyword combinations						
Academic Entrepreneurial Engagement	Academics Entrepreneurial Engagement AND Motivation	Academic Entrepreneurial Engagement AND Opportunity	Academic Entrepreneurial Engagement AND Ability	Frugal Innovation	Frugal Innovation AND Academic Entrepreneurial Engagement	Frugal Innovation AND Academic Entrepreneurial Engagement AND Education/University/HEI/IHL

*Search strategy*

The strategy used to collect the papers was based on five major online databases. The online databases were selected based on their wide range of social science research. Table 2 presents the results based on all the sources mentioned above and keyword sets. A total of two papers were listed when we used the keyword ‘Academic Entrepreneurial Engagement’. When we searched for ‘FI’, 1,067 papers were retrieved. When we used the keywords ‘AEE’ AND ‘Motivation’, two papers were listed. However, no papers were retrieved when we used ‘AEE’ AND ‘Opportunity’ and ‘FI’ AND ‘AEE Ability’. Finally, ‘AEE’ AND ‘Ability’ listed one paper. After careful selection based on the inclusion and exclusion criteria described above, we identified five papers relating to AEE and FI.

**Table 2.** Summary of search results.

No	Online database	Keyword combinations						
		Academic Entrepreneurial Engagement	Academics Entrepreneurial Engagement AND Motivation	Academic Entrepreneurial Engagement AND Opportunity	Academic Entrepreneurial Engagement AND Ability	Frugal Innovation	Frugal Innovation AND Academic Entrepreneurial Engagement	Frugal Innovation AND Academic Entrepreneurial Engagement AND Education/University/HEI/IHLs
**1**	Emerald	1	0	0	0	199	0	0
**2**	ProQuest	0	2	0	0	403	0	0
**3**	Inderscience	0	0	0	0	49	0	0
**4**	Scopus	0	0	0	0	101	0	0
**5**	Science Direct	1	0	0	1	315	0	0
	**Total:**	**2**	**2**	**0**	**1**	**1067**	**0**	**0**

IHLs – Institution of higher learning; HEI – Higher education institutions.

### Stage 3. Extracting and synthesising data

**Figure 3.**
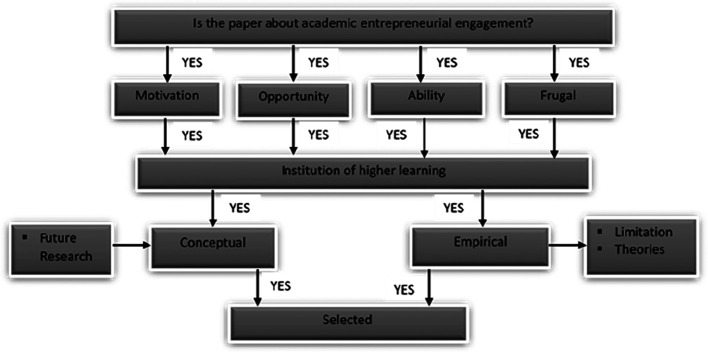
Extracting and synthesising process.

From the sources mentioned above, we extracted the papers based on the extraction process in Figure 3.

Figure 3 summarises the paper selection criteria for the review. From the databases and other sources, only AEE papers linked to motivation/opportunity/ability/FI/IHL were chosen for further review. The relevant papers based on the selection criteria are reported in the following subsections. From the 1,067 papers, we selected five for the final review.

Stages 4 and 5 of the Tranfield process are discussed in the following sections.

### Institutional Review Board Statement

Institutional Review Board Statement: Research Ethical Committee (REC) of Multimedia University (EA1392021). The study was conducted according to the guidelines and approved by the REC of Multimedia University, Cyberjaya, Malaysia.

## Results

### Summary of five core papers

Based on Tables 3 and 4, our findings revealed that no research was conducted in the area of FI for AEE related to motivation, opportunity and the ability of academics in PHEI. Most of the studies focused on factors shaping academics’ entrepreneurial intention, exploring individual, organisational and institutional variables. ^12^^,^^17^^,^^18^ The studies on academic entrepreneurial intention were conceptualised from different theoretical perspectives, including theory of planned behaviour (TPB), social learning theory and motivational theories. Based on TPB, academic attitude, subjective norms and behaviour control were associated with entrepreneurial intention. ^27^^,^^28^ Moreover, other factors such as perceived feasibility and desirability were determined to influence entrepreneurial intention, leading to actual behaviours. ^29^^,^^9^ Therefore, further research is necessary to explore the role of motivation, ability and AEE opportunity for FI.

**Table 3.** Summary of Five Core Papers.

Author (year)	Theory	Factors	Method	Challenges	Findings	FI	Motivation	Ability	Opportunity
^34^ Gümüsay & Bohné, (2018)	Resource oriented	1.Individual	Qualitative	Development of entrepreneurial competencies	Classification of relational, structural, and cultural cognitive inhibitors			✓	
		2. Contextual							
^30^ Halilem et al. (2017)	Multilevel approach		Quantitative	Academic entrepreneurial behavior	Copyright and income sharing schemes		✓		
^32^ Sulaimon et al. (2016)	Resource based view	Socio-economic characteristics	Quantitative	Commercializing research output process without violating extant rules	Academic responsibilities and entrepreneurial development are related				
		Dependent and independent variables							
^33^ Wang et al. (2020)	1. Social cognition	1. Individual	Quantitative	Determinants of academic entrepreneurial intentions	Previous commercialization experience		✓		
	2. Planned behavior	2. Organizational			Entrepreneurial support policies				
					Organizational scientific reputation				
					Individual academic output				
^31^ De Silva (2012)			Qualitative and quantitative	Entrepreneurial engagement in resource constrain environment	Lack of resources not a barrier				
					Engage in unrelated				
					Diversification				
					Being resource rich				

**Table 4.** Plotting of papers on FI, motivation, ability and opportunity.

Authors (year)	Frugal Innovation	Motivation	Ability	Opportunity
^34^ Gümüsay & Bohné (2018)			✓	
^30^ Halilem et al. (2017)		✓		
^32^ Sulaimon et al. (2016)				
^33^ Wang et al. (2020)		✓		
^31^ De Silva (2012)				

### Challenges and factors

*Challenges*

Several challenges constrain academic engagement in entrepreneurial activities. Such challenges were categorised as individual factors, which constrained personal engagement; organisational factors, which were structural and systemic inhibitors of engagement; and process factors, which included institutional policies detrimental to academic entrepreneurial behaviour.

Based on Table 5, existing studies highlighted the need for academics to assume job responsibilities beyond teaching and research as a major challenge to their engagement in entrepreneurial activities. ^13^ Academics are expected to extend their research output to products and services suitable for the industry and society. Thus, their research outcomes should be commercialised by creating new firms without breaching current laws that govern their role within the university. ^30^

**Table 5.** Extracting and synthesising process.

Challenges category	List of challenges (from Table 4)	Authors
Individual challenges	Academic entrepreneurial behavior	^30^ Halilem et al. (2017)
	Determinants of academic entrepreneurial intention	^33^ Wang et al. (2020)
	Entrepreneurial engagement in resource constrained environments	^31^ De Silva (2012)
Organization challenges	Development of entrepreneurial competencies	^34^ Gümüsay & Bohné (2018)
Process challenges	Commercializing research output process without violating extant rules	^32^ Sulaimon et al. (2016)

Academics are challenged to develop the appropriate competencies to become entrepreneurial to help create a firm. Competence refers to the academic ability to identify resources to start a business. ^31^ In addition, understanding the dynamics shaping the development of entrepreneurial competencies can improve capabilities to maximise resource use through FI, which means ‘accomplishing more with less’, thereby resulting in increased economic and societal value whilst reducing resource consumption. ^23^

*Factors*

Previous studies determined the relevant factors related to AEE. Regarding socioeconomic characteristics, gender, age, income and personality (proactiveness and optimism) were associated with academic entrepreneurial behaviour. ^32^ Drawing on TPB, Wang *et al*. ^33^ investigated the role of academic output and prior experience as individual factors and university reputation and supportive climate as organisational factors influencing AEE. ^34^ identified cultural factors as contextual inhibitors of academic competencies for entrepreneurial engagement. These studies are further summarised in Table 6.

**Table 6.** Factors influencing AEE.

Authors	List of factors
^32^ Sulaimon et al. (2016)	socio-economic characteristics
^33^ Wang et al. (2020)	Individual factors
	Organizational factors
^34^ Gümüsay & Bohné (2018)	Contextual factors

## Discussion

The discussions are described in Table 7 to answer the research questions as shown below.

**Table 7.** Answering research questions.

**Research gaps in studies on FI for AEE pertaining to motivation, opportunity and ability of academics in PHEI**
Based on Tables 3- 6, our findings revealed that little research was conducted in the area of FI for AEE related to motivation, opportunity and the ability of academics in PHEI. The mapping in Table 4 clearly indicates the scarcity of research on FI, motivation, opportunity and ability.
**Challenges faced by academics and key factors influencing pursuit of entrepreneurial initiatives in PHEI**
Based on the literature review of the various factors and challenges faced by academics in pursuing entrepreneurial initiatives in PHEI, we emphasised that attention should be paid to factors determining AEE, as few researchers examined the challenges faced by academics in developing the appropriate entrepreneurial competencies. The adoption of an FI mindset in AEE can create an opportunity to mitigate the identified challenges. FI minimises resource consumption and costs to attain high productive outcomes.
**FI as a game changer for PHEI to improve AEE**
AEE is a crucial factor, as PHEI must utilise their internal capabilities to promote FI through idea generation and relate them to their innovation ecosystem environment and the overall institutional structure. The scientific and technical competencies of universities will foster FI through the efficient use of resources under constrained environments and enable them to venture beyond the local market. Therefore, apart from their traditional mission and norms, PHEI that can realign their strategies to focus on sustainable societal development and address societal problems through academic research can enable FI. To develop FI in PHEI, AEE across various university community dimensions (academicians, scholars, faculty or the university) and collaboration with external stakeholders are necessary. Hence, PHEI will be able to integrate social, FI and environmental objectives through open innovation strategies via knowledge transfer and collaboration to produce patents, copyrights, intellectual property and spinoffs and startups with industries, business associations, the government, NGOs and communities rather than merely focusing on traditional financial objectives. As a result, PHEI must aim to promote teaching, research and community outreach activities that focus on achieving societal goals and contributing to FI to support AEE.

Limitation of this study is on the number of keywords selected. Keywords selections are based on research focus. However, there is possibility of obtaining more articles if the keywords are expanded to field of study that are not specific in nature such as intention. This could possibly be publication bias.

### Future research

We recommend future research to consider the following topics:

• AEE factors involving the COVID-19 MOC and other related aspects
• AEE for FI in PHEI focusing on adult learners
• AEE using qualitative methods that may unveil in-depth knowledge within this context based on actual academic entrepreneur case studies

## Conclusion

The purpose of this study is to notify academic entrepreneurial communities of the disparity in the implementation of FI to enhance AEE in studies published in the past 20 years. Our paper is based on an extensive review of the literature and the application of a methodology that includes five stages. This methodology is used to determine the scope and nature of FI to promote AEE. Despite the sizeable search result of 1,067 papers in the AEE domain, our search record is limited to five papers investigating FI for AEE. Based on our rigorous review of the five papers, we recommend further comprehensive research to determine whether FI is related to AEE and identify the challenges and factors supporting FI for AEE in PHEI and additional empirical work.

PHEI that can achieve FI will be able to enhance their AEE and contribute to the economy and social development, thereby creating IR 4.0 educational ecosystems.

## Data availability

Figshare. Excel file- Dataset.xlsx. DOI: https://doi.org/10.6084/m9.figshare.14872554.v1. ^35^

Figshare. Guideline Checklist

DOI: https://doi.org/10.6084/m9.figshare.16682626.v2. ^36^

This project contains the following data:

- This dataset is analysed for theories, type of papers, Academic entrepreneurial engagement factors and challenges, method and findings

Data are available under the terms of the Creative Commons Attribution 4.0 International license (CC-BY 4.0).
