# Supplementary material for: Academic entrepreneurial engagement for frugal innovation in higher education institutions: a systematic literature review
Source: F1000Res. 2023 May 16;10:1078. Originally published 2021 Oct 25. [Version 3] doi: 10.12688/f1000research.73312.3 (PMC10427802; doi:10.12688/f1000research.73312.3)
Supplement: Peer review history

# Peer review: Reviewer response for version 3

**DOI:** 10.5256/f1000research.147668.r191498

**Published:** 2023-08-15

**Authors:** Alessandro Muscio

**Affiliations:** 1 University of Foggia, Foggia, Italy

The paper deals with an original and interesting topic. However, it is flawed in many respects. First of all, the introduction is much larger than the other sections, including the literature review. Secondly, the discussion of the results is superficial. Third, it lacks of focus. Therefore, I must recommend its indexing only after major revisions.

 Comments:

- Since the paper is focused on FI in AEE, I would redraft the introduction accordingly. At the moment, it is largely focused on AEE. Moreover, the authors shift form AEE to FI without mentioning innovations. For me, the focus should be on academic R&I and on FI.
- Why the focus on private institutions? It might be relevant for Malaysia, but it is definitely not for the majority of other countries. At least there might be a difference in meanings. Please put this limitation into context and discuss better the generalizability of results to other countries.
- Why did the review omit students’ engagement? Scholars in many countries have concluded that students represent one of the most valuable source of AEE.
- What are the keywords used in the literature review? Notwithstanding the relevance of FI for academia, searching for “frugal innovation” might underestimate considerably the magnitude of the phenomenon. The “search strategy” section is not sufficiently clear.
- The implications and discussion are too superficial.
- In some cases I found references in footnotes, in others I found traditional citations. Please harmonise the style of citations.

Are the rationale for, and objectives of, the Systematic Review clearly stated?

Partly

Is the statistical analysis and its interpretation appropriate?

Not applicable

Are sufficient details of the methods and analysis provided to allow replication by others?

Partly

Are the conclusions drawn adequately supported by the results presented in the review?

No

Reviewer Expertise:

Academic engagement, academic entrepreneurship, technology transfer, innovation.

I confirm that I have read this submission and believe that I have an appropriate level of expertise to confirm that it is of an acceptable scientific standard, however I have significant reservations, as outlined above.

# Peer review: Reviewer response for version 3

**DOI:** 10.5256/f1000research.147668.r173879

**Published:** 2023-05-17

**Authors:** Murali Sambasivan

**Affiliations:** 1 Thiagarajar School of Management, Madurai, Tamil Nadu, India

This version is acceptable.

Are the rationale for, and objectives of, the Systematic Review clearly stated?

Yes

Is the statistical analysis and its interpretation appropriate?

Not applicable

Are sufficient details of the methods and analysis provided to allow replication by others?

Yes

Are the conclusions drawn adequately supported by the results presented in the review?

Yes

Reviewer Expertise:

NA

I confirm that I have read this submission and believe that I have an appropriate level of expertise to confirm that it is of an acceptable scientific standard.

# Peer review: Reviewer response for version 2

**DOI:** 10.5256/f1000research.79999.r159933

**Published:** 2023-02-03

**Authors:** Hasniyati Hamzah

**Affiliations:** 1 Department of Real Estate Faculty of Built Environment, Universiti Malaya, Kuala Lumpur, Malaysia

**Summary of review**

 Overall, the research topic is impactful in ensuring the sustainability of Malaysian private higher education institutions (PHEIs) by exploring academic entrepreneurial engagement (AEE) to generate institutional income. The problem statement was aptly formulated as it reflects the current difficulty faced by higher education institutions in Malaysia, and perhaps worldwide. The methodology was fairly explained, with some gaps that need to be addressed to increase the validity of the findings. Table 7 was useful in illustrating how the results of the SLR answer the research questions.

**Major issues**

1. It was not explained how the **1,067** papers were reduced to only **five** for the final review. The keywords in Table 1 did not differentiate between private and public HEIs. It was not known if the five final papers were from **private **higher education institutions' perspective. If no, how can the five final papers be used in the present study?
2. There was no explanation on the data extraction and analysis protocol. How many researchers were involved in the process? For more complex papers, how were biases, omissions and misinterpretations minimised to ensure an accurate understanding of the papers' theories and findings?

**Minor issues**

1. Proofreading: There are some grammatical/syntax oversights. E.g:
  - "In particular, we aim **to achieve the provide answers** to the following questions;" . Please check the bold phrase.
  - "...various challenges that hinder **then** in engaging..." Them?
2. There was no justification of the selection of only peer-review papers in this study. Could books and postgraduate thesis also add to the literature? There could be a good reason why only peer-review papers were considered and I'm interested to know the answer.

The above issues did not seem insurmountable and I hope that the authors will be able to either respond satisfactorily or amend the paper accordingly. 

 Thank you.

Are the rationale for, and objectives of, the Systematic Review clearly stated?

Yes

Is the statistical analysis and its interpretation appropriate?

Partly

Are sufficient details of the methods and analysis provided to allow replication by others?

Partly

Are the conclusions drawn adequately supported by the results presented in the review?

Partly

Reviewer Expertise:

Entrepreneurial education in real estate

I confirm that I have read this submission and believe that I have an appropriate level of expertise to confirm that it is of an acceptable scientific standard, however I have significant reservations, as outlined above.

# Response

**Published:** 2023-04-30

**Authors:** Magiswary Dorasamy

**Affiliations:** MULTIMEDIA UNIVERSITY, Malaysia

Dear Respected Reviewer,

 Thank you for very valuable comments. We have corrected the paper based on comments as per the following feedback: 

 1.How can the five final papers be used in the present study?

- The Prisma diagram shows the reduction of papers from 1067 to final five papers given in Fig.4 (which was missing in the original article).

2. There was no explanation on the data extraction and analysis protocol.

- Data extraction process was given in stage 3 extracting and synthesising data. The analysis involves basic descriptive information.

3. How many researchers were involved in the process?

- Two researchers was involved in keyword search of the literatures.

4. For more complex papers, how were biases, omissions and misinterpretations minimised to ensure an accurate understanding of the papers' theories and findings?

- The papers are straight forward and was easy to categorize as it appears clearly in the paper. Hence, there was no bias or misinterpretation issue.

5. There was no justification of the selection of only peer-review papers in this study.

- The justification is now added. See stage 2, first paragraph.

6. Could books and postgraduate thesis also add to the literature?

- Due to accessibility issue, we have not included books and postgraduate thesis.

# Peer review: Reviewer response for version 2

**DOI:** 10.5256/f1000research.79999.r122607

**Published:** 2022-02-28

**Authors:** Murali Sambasivan

**Affiliations:** 1 Thiagarajar School of Management, Madurai, Tamil Nadu, India

Revisions are acceptable. The authors have addressed the queries.

Are the rationale for, and objectives of, the Systematic Review clearly stated?

Yes

Is the statistical analysis and its interpretation appropriate?

Not applicable

Are sufficient details of the methods and analysis provided to allow replication by others?

Yes

Are the conclusions drawn adequately supported by the results presented in the review?

Yes

Reviewer Expertise:

NA

I confirm that I have read this submission and believe that I have an appropriate level of expertise to confirm that it is of an acceptable scientific standard.

# Peer review: Reviewer response for version 1

**DOI:** 10.5256/f1000research.76955.r97934

**Published:** 2021-11-23

**Authors:** Murali Sambasivan

**Affiliations:** 1 Thiagarajar School of Management, Madurai, Tamil Nadu, India

The article ‘ **Academic entrepreneurial engagement (AEE) for frugal innovation (FI) in higher education institutions: a systematic literature review**’ highlights the need for private higher educational institutions (PHEI) to move beyond teaching and research to overcome the current challenges such as COVID-19 pandemic, shrinking resources and so on. Specifically, the article deals with the systematic literature review related to engagement in entrepreneurial activities by academics through frugal innovation (FI). The article concludes that FI for AEE in PHEI can be a game-changer for future sustainability. 

 The theme of the research ‘the idea of academic entrepreneurial engagement leading to innovation,  identification of opportunities for new business ventures, financial rewards for institutions and academics, impact on the economy, and enhancement of social welfare ’ is interesting and requires further empirical research. This idea needs to be explored in both public and private higher learning institutions. 

 I am highlighting below a few points that can be addressed to enhance the quality of the paper. 

**Major Points**: 

- The introduction section must motivate the reader to read the complete article. Therefore, typically, the introduction section must address four questions: What do we know? What else do we need to know? Why do we need to know? What will be done in this research? The authors must revisit introduction based on these questions.
- Why frugal innovation? Why not just, innovation?
- Introduction must explicitly state the theoretical and practical contributions of this research.
- Research questions and objectives are same. It is sufficient to have questions or objectives, unless they are different.
- Why were motivation, opportunity, and ability used as keywords? Probably the keywords limited the number of papers selected.
- Discussion section must be elaborated with more references and examples. I hardly see, any. 

**Minor point: **

- To reedit the manuscript. 

Are the rationale for, and objectives of, the Systematic Review clearly stated?

Yes

Is the statistical analysis and its interpretation appropriate?

Not applicable

Are sufficient details of the methods and analysis provided to allow replication by others?

Yes

Are the conclusions drawn adequately supported by the results presented in the review?

Yes

Reviewer Expertise:

Management Science, Operations and Supply Chain Management, General Management

I confirm that I have read this submission and believe that I have an appropriate level of expertise to confirm that it is of an acceptable scientific standard, however I have significant reservations, as outlined above.

# Response

**Published:** 2021-11-30

**Authors:** Magiswary Dorasamy

**Affiliations:** MULTIMEDIA UNIVERSITY, Malaysia

Review Comment: The introduction section must motivate the reader to read the complete article. Therefore, typically, the introduction section must address four questions: What do we know? What else do we need to know? Why do we need to know? What will be done in this research? The authors must revisit introduction based on these questions.

 The introduction section must motivate the reader to read the complete article. Therefore, typically, the introduction section must address four questions: What do we know? What else do we need to know? Why do we need to know? What will be done in this research? The authors must revisit introduction based on these questions.

 Response: Thank you for your comment. The introduction has been revised as suggested. Please see paragraph1, page 2-4

 Reviewer Comment: 

 Why frugal innovation? Why not just, innovation?

 Response: Thank you for your comment. The PHEI is facing resource constraints especially budget constraints. As such, frugal innovation will help PHEI to accomplish more with less. Please see paragraph 3, page 4

 Reviewer Comment:

 Introduction must explicitly state the theoretical and practical contributions of this research.

 Response: Thank you for your comment. The theoretical and practical contributions have been addressed in the introduction section.

 Reviewer Comment:

 Research questions and objectives are same. It is sufficient to have questions or objectives, unless they are different

 Response: Thank you for your comment. We have retained the questions and removed the objectives.

 Reviewer Comment:

 Why were motivation, opportunity, and ability used as keywords? Probably the keywords limited the number of papers selected.

 Response: Our emphasis is to identify how AEE by way of motivation, opportunity and ability so that PHEI can achieve its goals (please see paragraph 2, page 4)

 Reviewer Comment:

 Discussion section must be elaborated with more references and examples. I hardly see, any.

 Response: Thank you for the comments. We did not cite the citations in this section as the discussion referred to the tables which contain the reference.
